# Pyroxene Ceramics Fabricated from Tailings via Synergistic Oxidation of Converter Slag and Copper Slag: Sintering Behavior, Microstructure Evolution and Mechanical Performance

**DOI:** 10.3390/ma19183836

**Published:** 2026-09-09

**Authors:** Hui Lin, Bowen Cao, Xuefei Zhang, Jiawei Wang, Xiaohui Huang, Min Chen, Nan Wang

**Affiliations:** 1 College of New Energy and Materials, Ningde Normal University, No. 1, Xue-Yuan Road, Ningde 352100, China; linhui@ndnu.edu.cn (H.L.);; 2 School of Metallurgy, Northeastern University, 3-11 Wen-Hua Road, Shenyang 110819, China

**Keywords:** converter slag, copper slag, synergistic treatment, tailings-derived ceramics, sintering, phase transformation, microstructure, properties

## Abstract

**Highlights:**

- Pyroxene-based ceramics are fabricated using tailings from synergistic oxidation of converter slag and copper slag.
- Clarified effect of tailings content and sintering conditions on ceramic sintering and performances.
- The tailings addition leads to a highly dense structure with more uniform and smaller pores.
- Leaching toxicity of the ceramics is significantly lower than the initial tailings.

**Abstract:**

Converter slag and copper slag represent promising sources for ceramic materials, but their high total iron content leads to waste of iron resources and inferior ceramic performance. To address this issue, we propose a two-step utilization method for converter slag and copper slag, including iron extraction synergistic oxidation and ceramic fabrication for tailings. In this study, the effects of tailings content, sintering temperature, and sintering aid addition on the phase composition, microstructure, physico-mechanical properties, and leaching characteristics of the novel pyroxene-based ceramics were investigated. The results reveal that the ceramics containing 50 wt% tailings present a single pyroxene phase with uniformly dispersed fine closed pores. At a sintering temperature of 1190 °C, the optimized ceramic achieves a water absorption of 0.33% and a flexural strength of 80.5 MPa. Elevating the sintering temperature facilitates the grain growth of pyroxene crystals and the formation of a liquid phase. The addition of sintering aid effectively lowers the sintering temperature and improves the densification degree of ceramic matrices. In addition, the leaching toxicity of the prepared ceramics is well below the standard regulatory limits. This study provides a novel approach for the high-value recycling of tailings.

## 1. Introduction

Converter slag is a predominant solid waste generated in the steelmaking industry, with a global annual output exceeding 100 million tonnes. Its chemical composition mainly consists of 40–50% CaO, 5–20% SiO_2_, 15–26% TFe, 2–15% MgO, and 1–6% MnO [1,2,3,4,5]. At present, most steel plants still dispose of converter slag via landfilling or stockpiling, resulting not only in wastage of valuable iron resources but also posing potential environmental risks. To address these issues, extensive studies have been devoted to the reduction and oxidative modification of converter slag through the addition of siliceous modifiers [6,7,8,9], aiming to achieve efficient and deep recovery of iron resources.

Copper slag is the primary by-product from copper smelting, and the global annual output is estimated to exceed 40 million tons. Copper slag contains abundant valuable metallic elements including Fe and Cu [10,11], and current pyrometallurgical routes for recovering valuable components from copper slag include oxidation methods. However, iron recovery efficiency is inherently limited by the fact that iron in copper slag is predominantly locked in fayalite phases with a stable crystal structure [12,13]. To overcome this limitation, calcium-based modifiers are widely adopted to disrupt the stable fayalite network, which promotes the oriented formation of magnetite during oxidation [14,15,16,17].

Given the complementary chemical characteristics of converter slag (high calcium) and copper slag (high silicon), our previous work [18] proposed an innovative synergistic oxidation treatment process for the two metallurgical slags. By exploiting the interactive modification between the calcium source from converter slag and the silicon source from copper slag, the weakly magnetic iron-bearing phase in both slags can be synchronously converted into strongly magnetic MgFe_2_O_4_ spinel, which can subsequently recover via low-cost magnetic separation. Nevertheless, this synergistic process still produces a large amount of magnetic separation tailings with a typical CaO-MgO-Al_2_O_3_-SiO_2_ (CMAS) chemical system. Improper disposal of these tailings will pose severe environmental hazards. Therefore, the high-value hierarchical utilization of magnetic separation tailings derived from the synergistic treatment of multi-source metallurgical solid wastes is an urgent research topic that requires in-depth investigation.

Pyroxene-based ceramics are representative CMAS-system functional materials, which have attracted widespread attention due to their superior mechanical performance and excellent chemical corrosion resistance, showing broad application prospects in construction and industrial engineering fields [19,20,21]. At present, the commercial production of pyroxene ceramics still heavily relies on natural mineral raw materials and the over-exploitation of primary mineral resources has inevitably caused resource depletion and ecological degradation. Against this background, the fabrication of high-performance ceramics using industrial tailings and solid wastes has important practical value and strategic significance for resource recycling and carbon neutrality. Relevant exploratory studies have been reported in recent years. Ji et al. [22] prepared ceramics tiles with steel slag and waste clay bricks, and the primary crystalline phases of samples were akermanite, diopside, and magnetite. The obtained ceramics achieved flexural strength over 73.2 MPa and a water absorption rate below 0.05%. Zhao et al. [23,24,25] successfully developed a novel CMSA-system pyroxene ceramic using steel slag as the main raw material, and clarified the intrinsic correlation between phase composition and mechanical properties. A pyroxene phase was formed at 40 wt% steel slag addition, endowing the ceramics with optimal mechanical performance and a maximum flexural strength of 143 MPa. Zhang et al. [26] used copper slag, fly ash, and waste glass to fabricate glass-ceramics, and the results showed that increasing copper slag content enhanced the diffraction peak intensity of diopside and improved the mechanical properties of the composites.

Despite the above research progress, existing technical routes still have prominent limitations. First, the iron-rich phases in converter slag and copper slag degrade grindability, significantly increasing industrial grinding and production costs. More importantly, ceramic materials have stringent limitations on iron content; excess iron impurities will seriously impair the surface appearance, color uniformity and long-term mechanical stability of ceramic products [27]. In comparison, the magnetic separation tailings obtained from the synergistic treatment of converter slag and copper slag possess a CMAS composition that is highly compatible with pyroxene ceramic fabrication, with iron content drastically reduced after magnetic separation. Such tailings are therefore ideal low-cost raw materials for the preparation of high-quality pyroxene ceramics, exhibiting multiple inherent advantages. Firstly, the tailings can replace traditional calcium carbonate as the calcium source, eliminating the porous microstructure defects caused by carbonate thermal decomposition during sintering. Secondly, the substitution of natural minerals with metallurgical tailings reduces raw material costs, and provides a feasible disposal pathway for bulk metallurgical slag resources. Thirdly, the dense inherent microstructure of the tailings accelerates atomic diffusion and solid-phase reaction kinetics during firing, which enables the reduction in sintering temperature and production energy consumption.

In this work, novel high-performance pyroxene-based ceramics were fabricated using the aforementioned magnetic separation tailings as the primary raw material. The synergistic effects of tailings content, sintering temperature and sintering aid addition on the phase composition, microstructure evolution, and physical and mechanical properties of the as-prepared ceramics were systematically investigated. This study aims to elucidate the material formation mechanism of tailings-based pyroxene ceramics, and provide theoretical guidance and technical support for the high-value utilization of such metallurgical tailings in the ceramic industry. The findings are expected to promote the green transformation and sustainable development of both metallurgical and building material industries.

## 2. Materials and Methods

### 2.1. Materials

**Figure 1:**
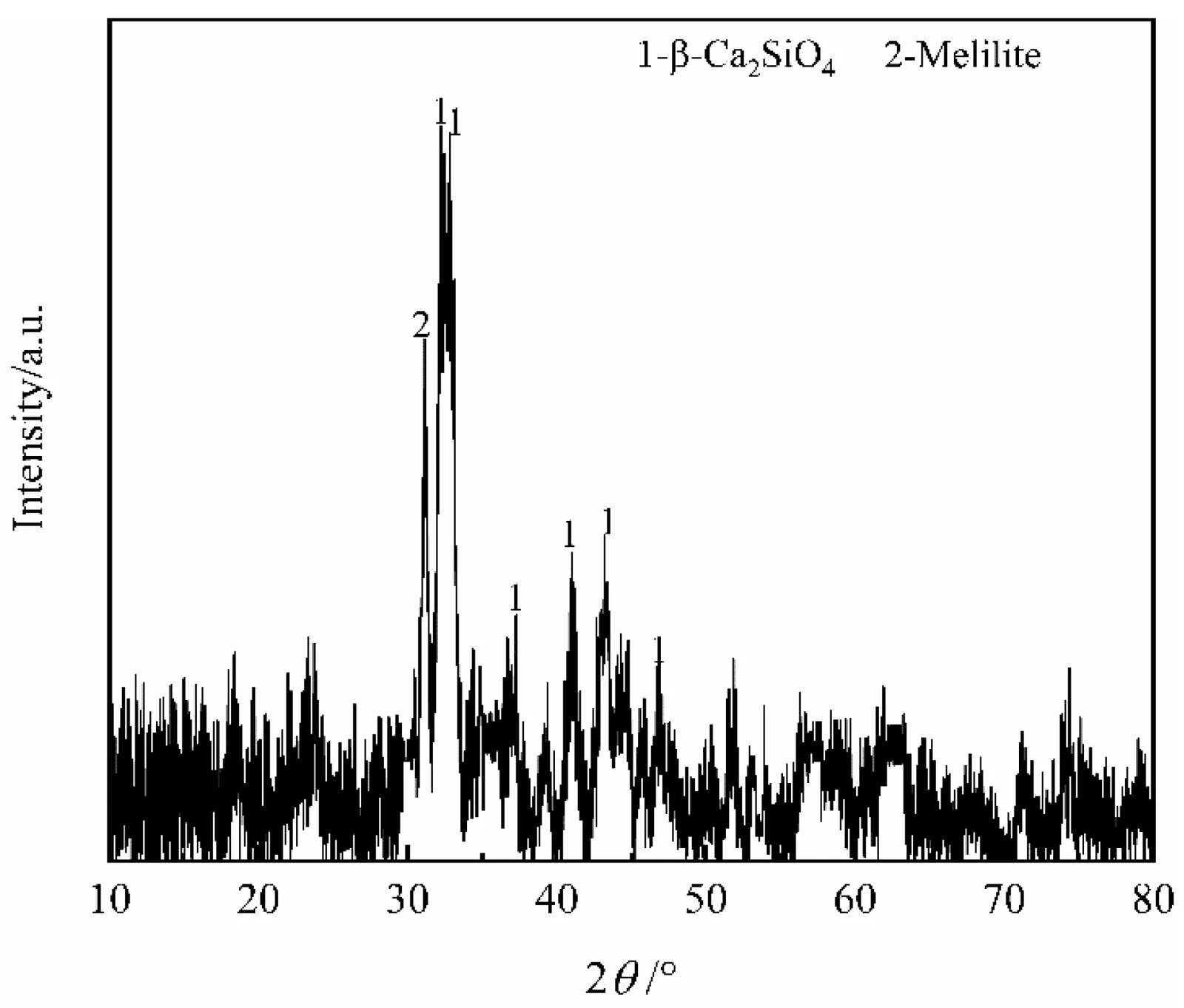
XRD pattern of the magnetic separation tailings.

The raw materials used in this experiment include magnetic separation tailings obtained from the synergistic oxidation treatment of converter slag and copper slag, which were collected from Anshan Steel Co., Ltd., Anshan, China and Zijin Copper Co., Ltd., Longyan, China respectively, along with conventional ceramic industrial raw materials, namely kaolin, quartz, talc, and albite (Shanghai Macklin Biochemical Co., Ltd., Shanghai, China). The chemical compositions of all raw materials were characterized via X-ray fluorescence (XRF) spectrometry (ZSX100e, Rigaku Corporation, Tokyo, Japan), and the corresponding results are summarized in Table 1. XRF results indicate that the magnetic separation tailings are predominantly CaO, SiO_2_, MgO and Al_2_O_3_, with high contents of CaO and SiO_2_, and relatively low contents of MgO and Al_2_O_3_. In the formulated ceramic system, kaolin provides SiO_2_ and Al_2_O_3_ for the ceramic matrix, while quartz serves as a pure SiO_2_ source. Due to the low MgO content in tailings, talc was selected as the primary Mg element source in this formula for fabricating pyroxene-based ceramics. To further reduce the sintering temperature of tailings-derived ceramics, albite was used as a fluxing agent in this work. Additionally, the phase composition analysis verified that the magnetic separation tailings mainly consist of crystalline phases of dicalcium silicate and melilite, as illustrated in Figure 1.

To clarify the effects of tailings content and sintering aid addition on the sintering behavior and ceramics properties, a series of ceramic formulations were designed. First, specimens with tailings dosages varying from 30 wt.% to 60 wt.% were fabricated and designated as S30, S40, S50, and S60. Furthermore, on the basis of the optimal tailings content of 50 wt.%, additional groups with albite addition amounts of 1 wt.%, 2 wt.%, and 3 wt.% were prepared and coded as NS1, NS2, and NS3, respectively. The detailed raw material formulations of all designed batches are summarized in Table 2 and Table 3.

### 2.2. Experimental Procedure

Raw materials of each group were wet-mixed in the planetary ball mill for 30 min to guarantee uniform homogenization. The obtained slurry was then dried at 120 °C for 24 h and sieved through a 200-mesh standard screen. The sieved mixtures were filled into a mold and uniaxially pressed into cylindrical samples (30 mm in diameter, and 10 mm in height) under 150 MPa pressure. Subsequently, according to the relevant literature on CMSA-system ceramics derived from solid wastes and preliminary exploratory sintering experiments, the green compacts were placed in a high-temperature electric furnace, heated at a rate of 8 °C/min to the designated sintering temperature, and soaked for 1 h before furnace-cooling to room temperature. The sintering temperature range in this experiment was 1150–1210 °C with an interval of 20 °C. Table 4 presents the chemical compositions of the as-prepared ceramics, which mainly include CaO, SiO_2_, MgO, and Al_2_O_3_.

### 2.3. Characterization

The phase compositions of the sintered ceramics were identified by X-ray diffraction (XRD, X’pert PRO, PANalytical, The Netherlands) with a scanning angle ranging from 10° to 80°. The microstructural morphology of ceramic specimens was characterized using field emission scanning electron microscopy (FEI, Hillsboro, OR, USA). The bulk density, water absorption and apparent porosity of the ceramic samples were measured by the Archimedes method, and the flexural strength of the fired samples was measured by a 3-point bending test on a universal testing machine (Chinese National Standard GB/T 6569-2006 [28]; span, 30 mm; crosshead speed, 0.5 mm/min; specimen size, 3 mm × 4 mm × 35 mm). Note that the specimens for the flexural strength test were cut from the rectangular sintered bars using a diamond saw. In this work, at least five parallel specimens were tested for each group, and the test results were characterized by the mean value ± standard deviation to represent the variability of the measurements. All invalid specimen data where fracture occurred outside the indenter loading area were excluded and not included in the statistical calculations. The calculation method is as follows:

(1) $$R=\frac{3FL}{2bh^{2}}$$

*R* is the flexural strength of the specimen, MPa; *F* is the fracture load, N; *L* is the span length, mm; *b* is the specimen width, mm; *h* is the minimum thickness of the specimen at the fracture edge, mm.

**Figure 2:**
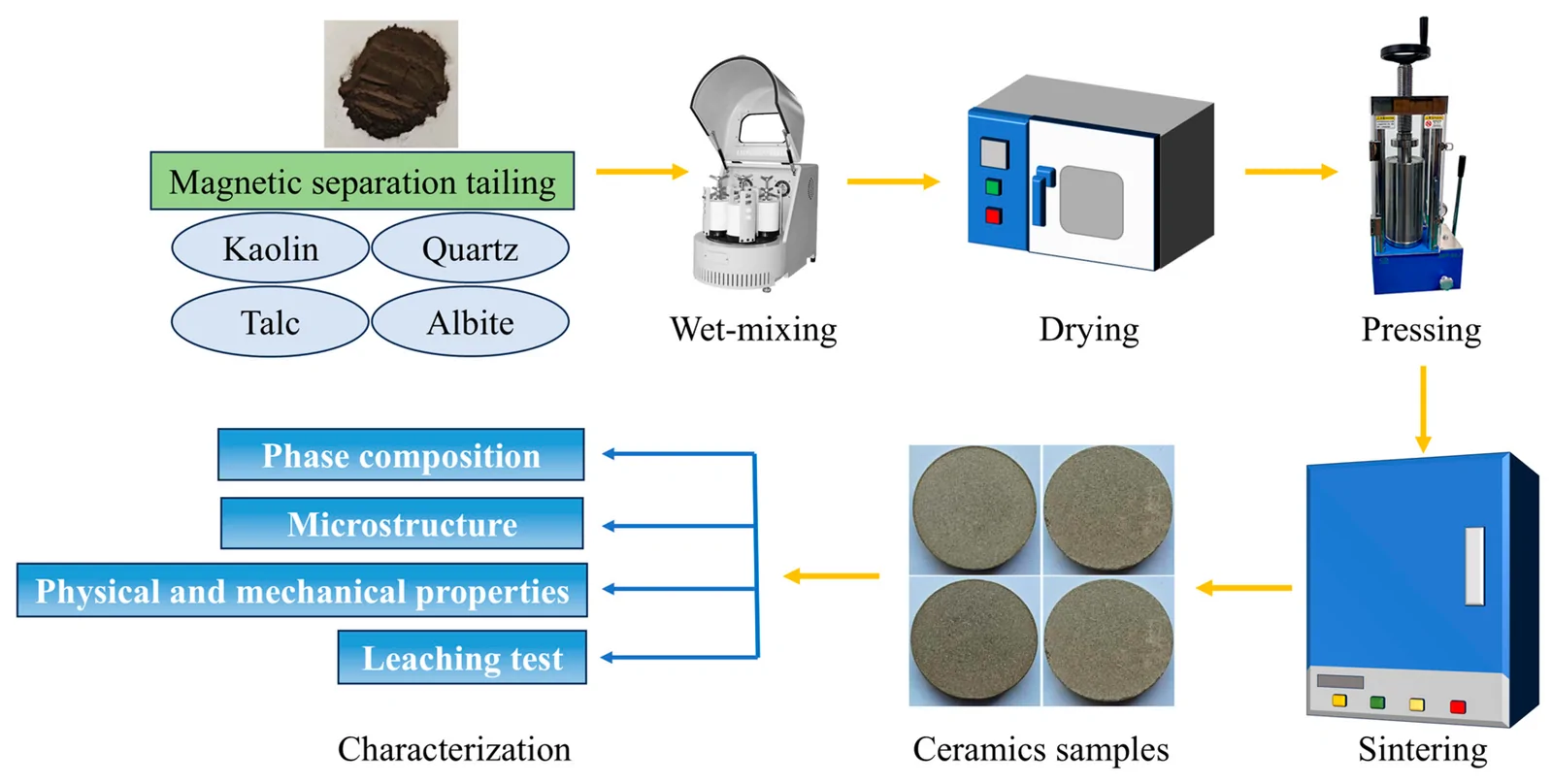
Flow chart for the ceramic sample preparation from magnetic separation tailings.

The environmental risk of the as-fabricated ceramics was evaluated according to the Chinese Standard HJ/T 557-2010 [29]. A horizontal vibration method was adopted to conduct heavy metal leaching tests with a solid–liquid ratio of 1:10 (kg/L), an oscillation frequency of 110 ± 10 cycles per minute, and a vibration amplitude of 40 mm. After 16 h of continuous leaching, the resultant leachate was collected, and the concentrations of toxic elements were determined by inductively coupled plasma atomic emission spectrometry (ICP-AES). The overall fabrication and characterization workflow of pyroxene-based ceramics derived from magnetic separation tailings is illustrated Figure 2.

## 3. Results and Discussion

### 3.1. Phase Transformation of Tailings Pyroxene-Based Ceramics

#### 3.1.1. Effect of Tailings Content

**Figure 3:**
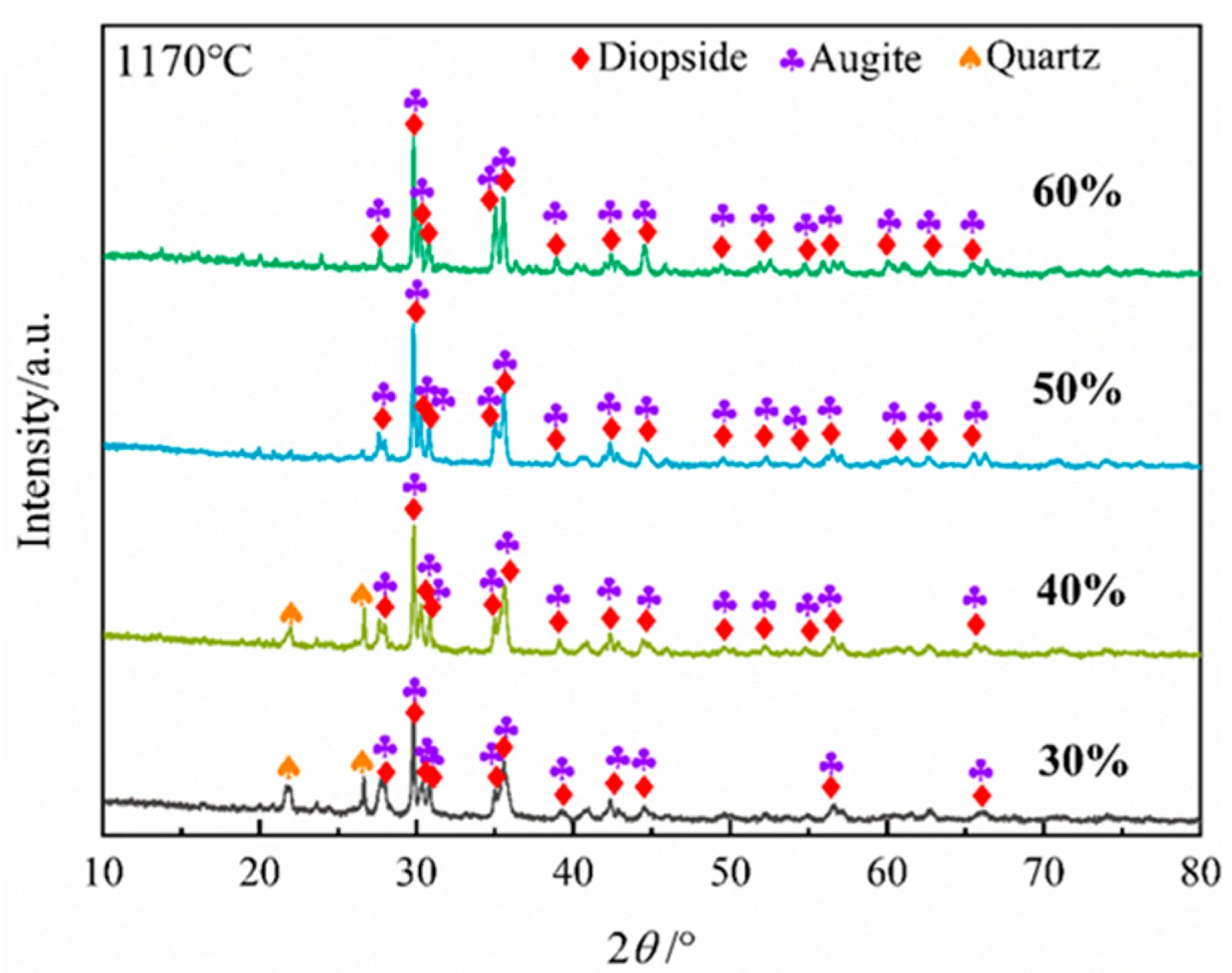
XRD patterns of the ceramics with various tailings contents.

Figure 3 presents the XRD patterns of samples S30, S40, S50 and S60 sintered at 1170 °C. It can be observed that the main crystalline phases of all samples are diopside and augite, both of which belong to the pyroxene mineral group, and the intensity of the pyroxene diffraction peaks gradually increases with increasing tailings content. Notably, residual quartz phase is detected in samples S30 and S40, accompanied by relatively weak pyroxene diffraction signals, which indicates an incomplete sintering reaction in these two samples. Tailings act as the main source of CaO in the system. At low tailings content, the supply of CaO within the system becomes insufficient. Consequently, quartz cannot be fully consumed during sintering and remains as a residual crystalline phase. This inhibits the nucleation and growth of the pyroxene phase, leading to weak diffraction peaks of pyroxene. Furthermore, quartz is susceptible to crystalline phase transformation during the cooling stage, which may exert adverse effects on the comprehensive performance of the fabricated ceramics. In comparison, no crystalline phases outside the pyroxene phase were detected in samples S50 and S60. The phase formation and transformation mechanisms are illustrated by the reaction Equations (2)–(6).

(2) $${\mathrm{Al}}_{2}O_{3}\cdot 2{\mathrm{SiO}}_{2}\cdot 2H_{2}O\left(s\right)={\mathrm{Al}}_{2}O_{3}\cdot {2\mathrm{SiO}}_{2}\left(s\right)+{2H}_{2}O\left(g\right)$$

(3) $$3\mathrm{MgO}\cdot {4\mathrm{SiO}}_{2}\cdot H_{2}O\left(s\right)={3\mathrm{MgSiO}}_{3}\left(s\right)+{\mathrm{SiO}}_{2}\left(s\right)+H_{2}O\left(g\right)$$

(4) $$2\mathrm{CaO}\left(s\right)+{\mathrm{MgSiO}}_{3}\left(s\right)+{\mathrm{SiO}}_{2}\left(s\right)={\mathrm{Ca}}_{2}{\mathrm{MgSi}}_{2}O_{7}\left(s\right)$$

(5) $$\mathrm{CaO}\left(s\right)+{\mathrm{MgSiO}}_{3}\left(s\right)+{\mathrm{SiO}}_{2}\left(s\right)={\mathrm{CaMgSi}}_{2}O_{6}\left(s\right)$$

(6) $${\mathrm{Ca}}_{2}{\mathrm{MgSi}}_{2}O_{7}\left(s\right)+{\mathrm{MgSiO}}_{3}\left(s\right)+{\mathrm{SiO}}_{2}\left(s\right)={2\mathrm{CaMgSi}}_{2}O_{6}\left(s\right)$$

Among the above reactions, Equations (2) and (3) describe the thermal decomposition of kaolin and talc raw materials, which produce metakaolin, enstatite, and quartz as decomposition products, respectively. The tailings provide the calcium source required for the synthesis of pyroxene-based ceramics. During the sintering process, akermanite and diopside are initially generated via the reactions described in Equations (4) and (5). Nevertheless, no characteristic diffraction peaks of akermanite are identified in the XRD patterns, demonstrating that akermanite acts as a transient intermediate phase. With a further rise in sintering temperature, the intermediate akermanite reacts with residual quartz and converts into diopside, as shown in Equation (6). Meanwhile, pyroxene is a general term for a mineral group with the general formula M_2_M_1_T_2_O_6_. According to relevant studies, extensive ionic isomorphous substitution may occur within this mineral group during sintering. Some metal cations (Al^3+^, Fe^3+^, Mn^3+^) may dissolve into the diopside crystal lattice, which in turn transforms partial diopside into augite. Therefore, based on the phase composition of the ceramics prepared in this study, it is inferred that the pyroxene phase consists of both diopside and augite.

#### 3.1.2. Effect of Sintering Temperature

**Figure 4:**
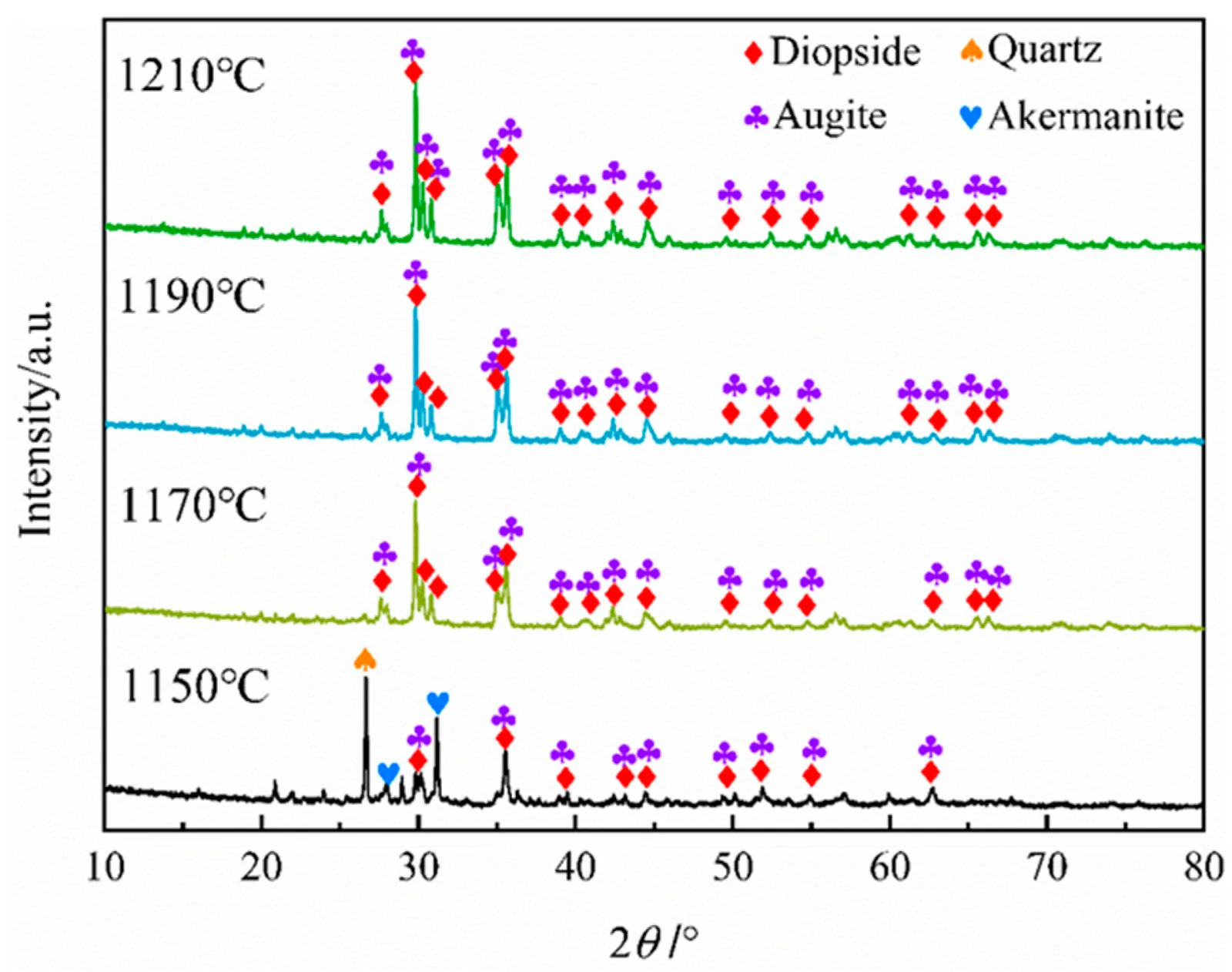
XRD patterns of the ceramics with various sintering temperatures.

Sintering temperature acts as a dominant factor governing the comprehensive performances of ceramic materials. In this work, phase assemblages of S50 specimens sintered at 1150–1210 °C were characterized, with the corresponding XRD results presented in Figure 4. At 1150 °C, quartz and akermanite constitute the primary crystalline phases, accompanied by weak diffraction signals of pyroxene, revealing an incomplete sintering reaction at this temperature. Upon elevating the temperature to 1170 °C and 1190 °C, the characteristic peaks of quartz and akermanite vanish completely. Meanwhile, pyroxene diffraction peaks are drastically intensified with narrowed peak widths. This phenomenon demonstrates that thermal elevation accelerates ionic diffusion and liquid-phase generation, which favors the nucleation and crystal growth of pyroxene and improves the densification of ceramic bulks. From 1150 °C to 1190 °C, XRD patterns show the gradual disappearance of quartz and akermanite peaks, accompanied by intensified and narrowed pyroxene (diopside-augite) diffraction peaks. The disappearance of impurity-phase peaks indicates that higher temperature promotes solid-state reactions and phase transformation, consuming intermediate akermanite and residual quartz. The increase in peak intensity together with peak-narrowing suggests enhanced crystallinity and crystal growth of pyroxene, which is consistent with accelerated ionic diffusion and liquid-phase-assisted crystallization upon heating. Moreover, the effects of sintering temperature on the crystal growth of the pyroxene phase and the physico-mechanical properties of ceramics will be discussed in Section 3.2.2 and Section 3.3.

Nevertheless, further increasing the sintering temperature to 1210 °C leads to a reduction in the peak intensity of the pyroxene phase. Such variation may stem from excessive liquid-phase formation. The abundant liquid phase readily wets and dissolves the newly formed pyroxene crystallites, thereby inhibiting the densification of the ceramic matrix.

#### 3.1.3. Effect of Sintering Aid Addition

**Figure 5:**
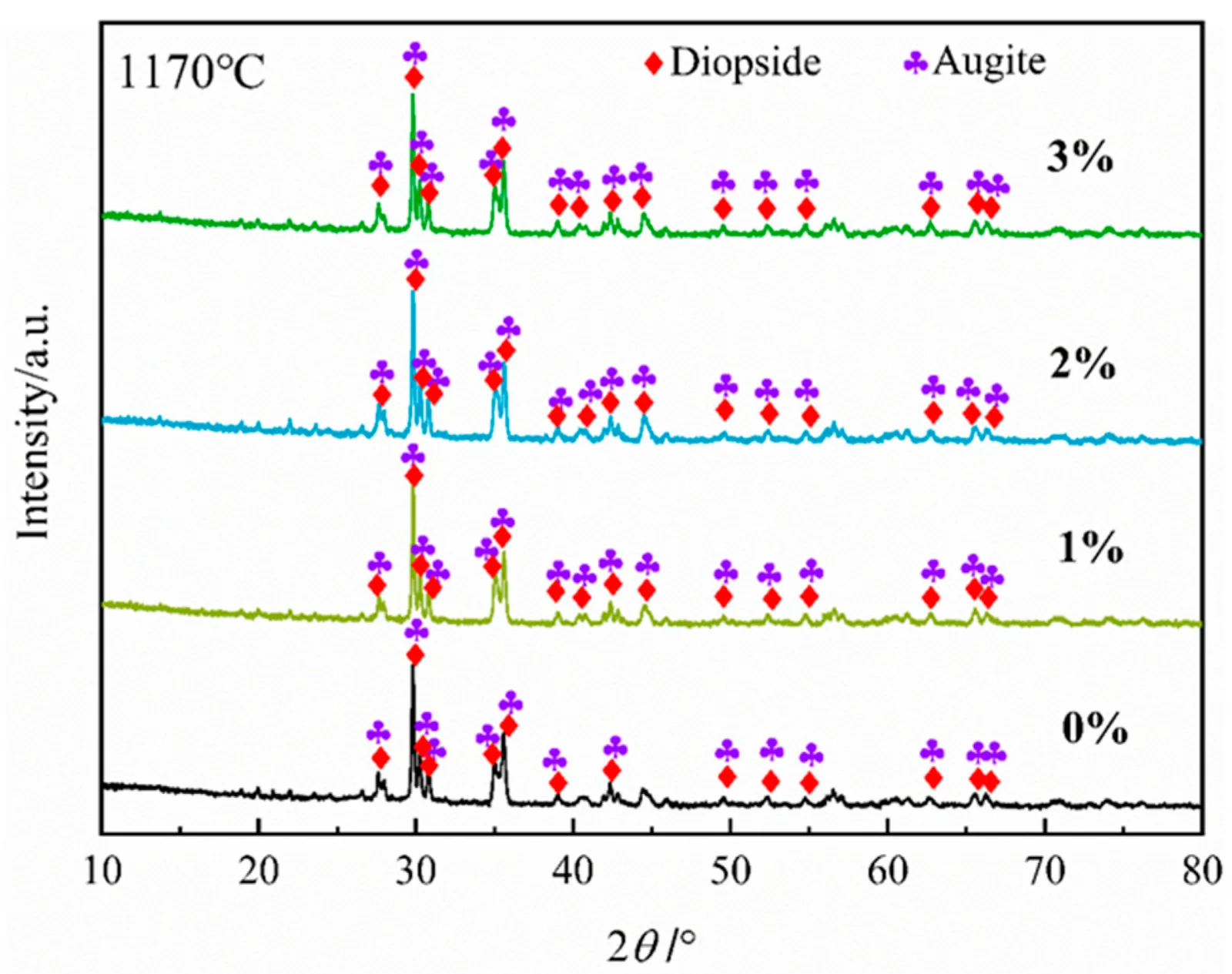
XRD patterns of the ceramics with various sintering aid additions.

Due to the high content of CaO and SiO_2_ in the tailings, ceramics prepared from tailings require a relatively high firing temperature. Albite can be added as a sintering aid to lower the firing temperature by relying on the alkali metal Na_2_O in albite to play a melting aid role. The addition of the sintering aid ranges from 1% to 3%. Figure 5 shows the phase composition of sample S50 with varying sintering aid additions at a sintering temperature of 1170 °C. As can be seen, compared with sample S50 without sintering aid, the samples containing the sintering aid consist of the pyroxene phase, and no significant difference is observed in the diffraction peak intensities. The alkaline metal Na_2_O in albite can act as a network modifier, reducing the connecting degree of silicon-oxygen bonds in the pyroxene phase and the polymerization degree of the melt, thereby promoting the formation of a liquid phase and the growth of pyroxene grains at a lower firing temperature. During the sintering process, the Na_2_O from albite either forms a Na^+^-bearing solid solution in the pyroxene phase or dissolves into the liquid phase, without forming any new phase [30].

### 3.2. Microstructure Evolution of Tailings Pyroxene-Based Ceramics

#### 3.2.1. Effect of Tailings Content

**Figure 6:**
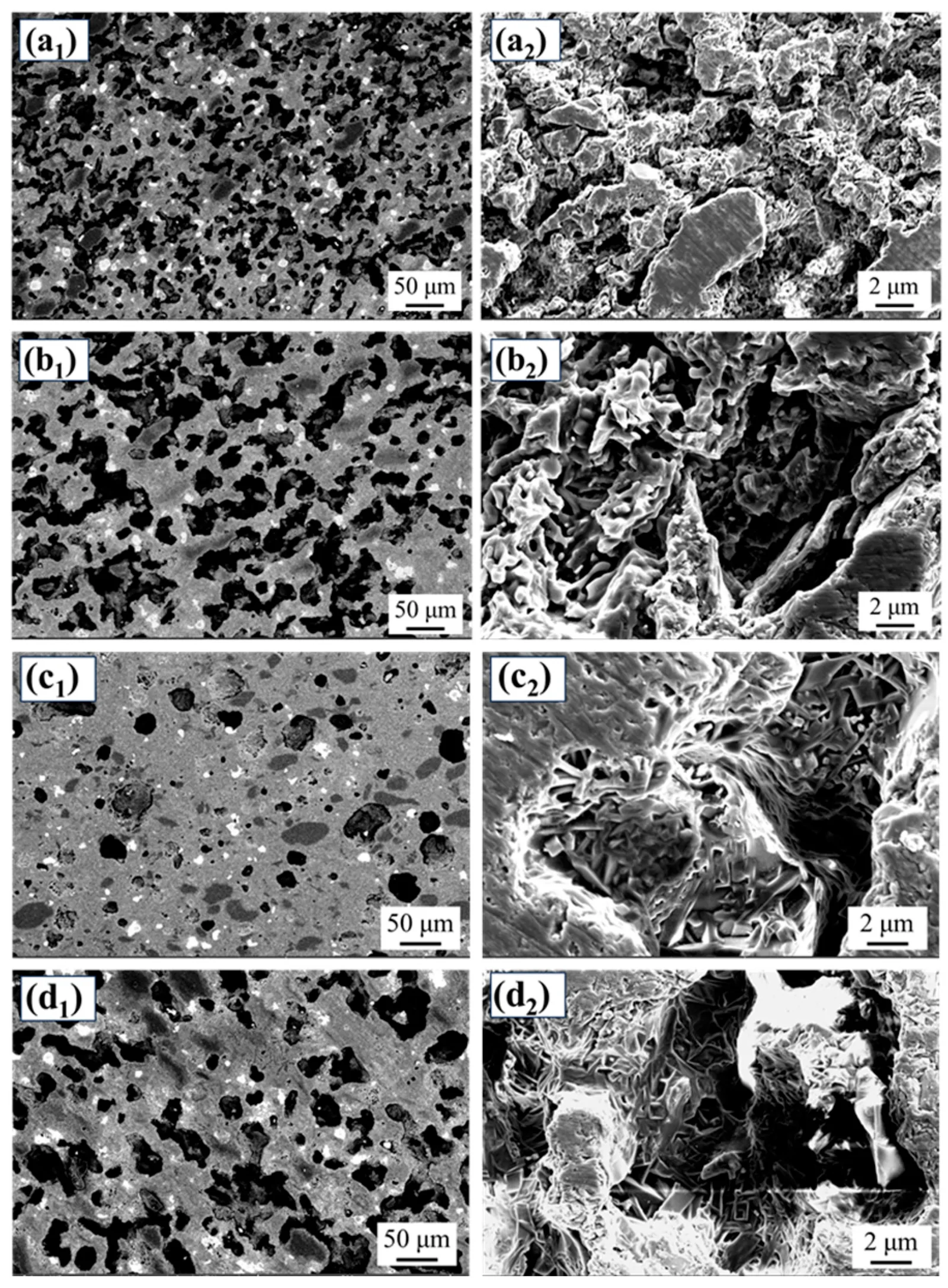
Microstructure of the ceramics with various tailings contents: (**a_1_**,**a_2_**) 30%; (**b_1_**,**b_2_**) 40%; (**c_1_**,**c_2_**) 50%; (**d_1_**,**d_2_**) 60%.

Figure 6 shows the microstructure of the ceramic samples sintered at 1170 °C with different tailings contents. As can be seen, the S30 and S40 samples present a loose and porous structure, and a large number of interconnected pores. The number and large size of the pores indicate insufficient liquid phase formation during the firing process and incomplete densification of the samples [31]. The S50 sample displays a more uniform pore distribution with a significant reduced number of pores, and the pore shape transforms from narrow and interconnected to approximately circular and closed pores. Furthermore, the pyroxene grains evolve from granular to rod-shaped and columnar. The crystals are distributed in a continuous network structure, which is favorable for promoting the densification of the ceramic matrix. In contrast, the microstructure of sample S60 reveals large and interconnected pores, uneven grain distribution, and a marked decrease in structural compactness. This is likely due to the fact that high tailings contents generate excessive liquid phase locally, and the liquid phase contracts rapidly during cooling. Therefore, the tailings content added to the ceramics should not exceed 50%.

#### 3.2.2. Effect of Sintering Temperature

**Figure 7:**
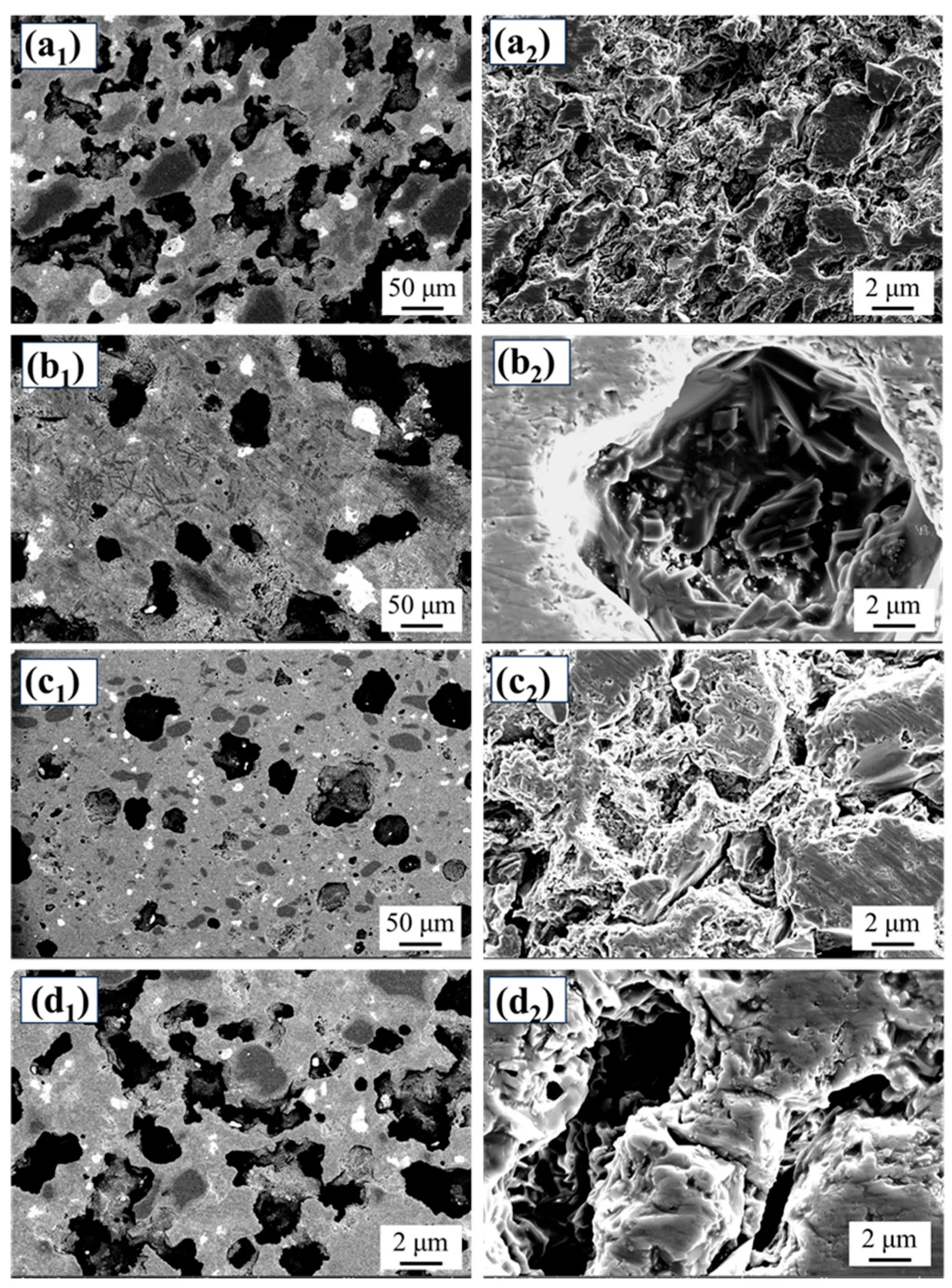
Microstructure of sample S50 with various sintering temperatures: (**a_1_**,**a_2_**) 1150 °C; (**b_1_**,**b_2_**) 1170 °C; (**c_1_**,**c_2_**) 1190 °C; (**d_1_**,**d_2_**) 1210 °C.

The formation of a liquid phase in ceramics determines the number and size of pores, thereby influencing the physical and mechanical properties of the ceramics. Sintering temperature is one of the main factors affecting the liquid phase formation. To investigate the effect of sintering temperature on the microstructure, the microstructure of the S50 ceramics sample was analyzed at varying sintering temperatures, and the results are shown in Figure 7.

At a sintering temperature of 1150 °C, abundant irregular interconnected pores are observed in the specimen, with the maximum size reaching nearly 100 μm. The ceramics possesses a loose interior and low densification degree, indicating that only a small volume of liquid phase forms at this relatively lower temperature, accompanied by sluggish ion diffusion, which is unfavorable for the elimination of internal pores. When the sintering temperature is raised to 1170 °C and 1190 °C, the number of pores drops drastically, and the residual pores gradually evolve into spherical closed pores. Meanwhile, the pyroxene grains exhibit a columnar and plate-like morphology, forming a compact interconnected network, which facilitates the improvement in mechanical properties of the ceramic specimens [32]. At 1210 °C, the pyroxene crystal phase melts to produce excessive liquid phase. Gas encapsulated within pores expands under continuous thermal exposure, which raises the porosity and disrupts the compact microstructure of the sample. Accordingly, the sintering temperature must be kept below 1210 °C.

#### 3.2.3. Effect of Sintering Aid Addition

**Figure 8:**
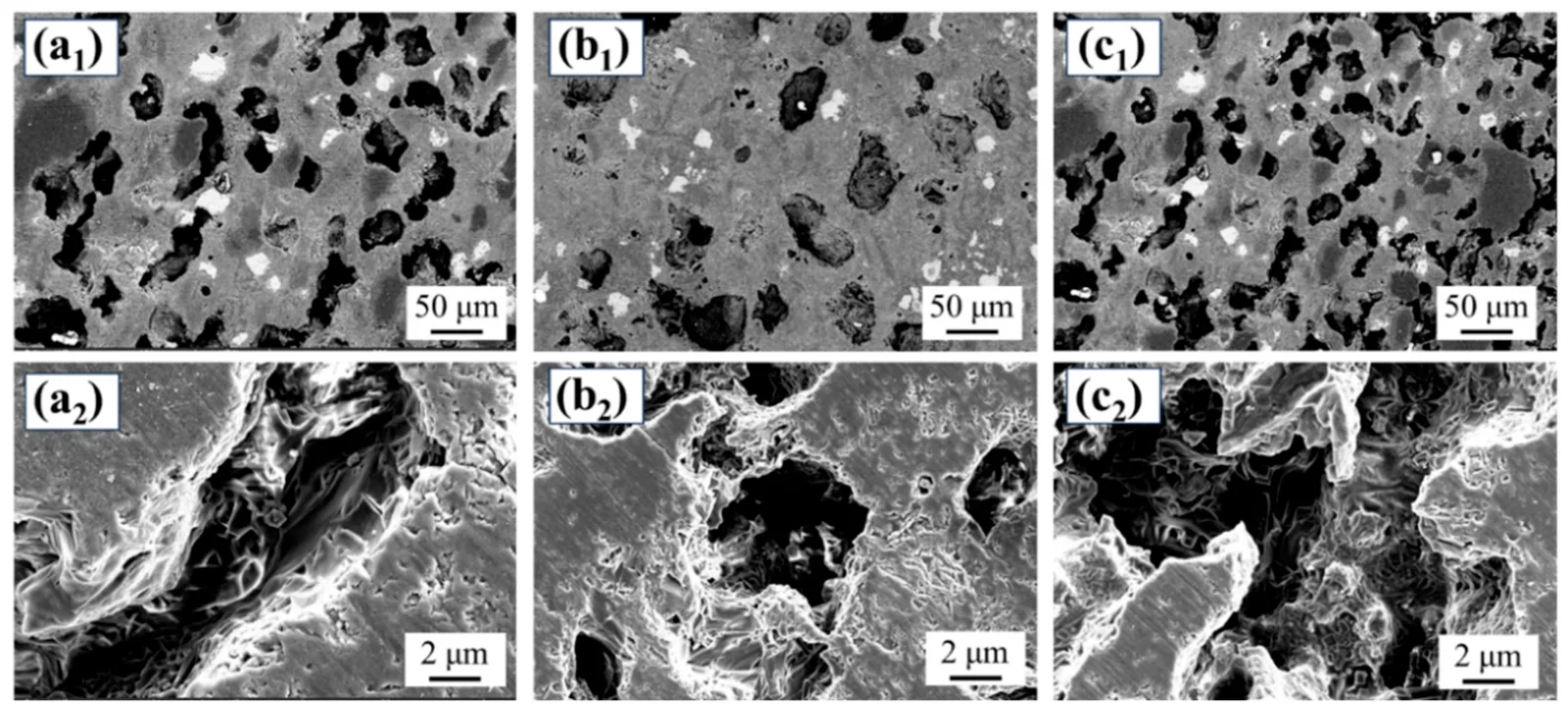
Microstructure of the ceramics with various sintering aid additions: (**a_1_**,**a_2_**) 1%; (**b_1_**,**b_2_**) 2%; (**c_1_**,**c_2_**) 3%.

As illustrated in Figure 8, increasing the sintering aid dosage from 1 wt% to 2 wt% drastically reduces the pore quantity inside ceramic specimens. Meanwhile, pores evolve into spherical shapes and the internal microstructure achieves higher densification. The incorporation of sintering aids facilitates liquid-phase formation, accelerates the generation of isolated closed pores, which exerts a positive effect on improving the densification of ceramics. However, further raising the sintering aid content to 3 wt% causes a dramatic growth in porosity. The sintered ceramic shows a distinct microstructure from the above samples when the sintering aid addition content increases to 3 wt%, as shown in Figure 8(c1,c2). The microstructure is very inhomogeneous due to the generation of many big pores in the matrix. As reported in the literature [33], this type of structure is attributed to a dramatic shrinkage in local regions during sintering due to the emergence of abundant liquid. In this case, grains lose their tight interlocking structure and display reduced grain sizes. An excess amount of sintering aid generates excessive liquid phase, which induces severe localized shrinkage of the matrix, and restricts complete grain growth. Moreover, oversized pores originating from surplus liquid phase give rise to intense stress concentration, degrading the overall comprehensive properties of the ceramics.

### 3.3. Physical and Mechanical Properties of Tailings Pyroxene-Based Ceramics

**Figure 9:**
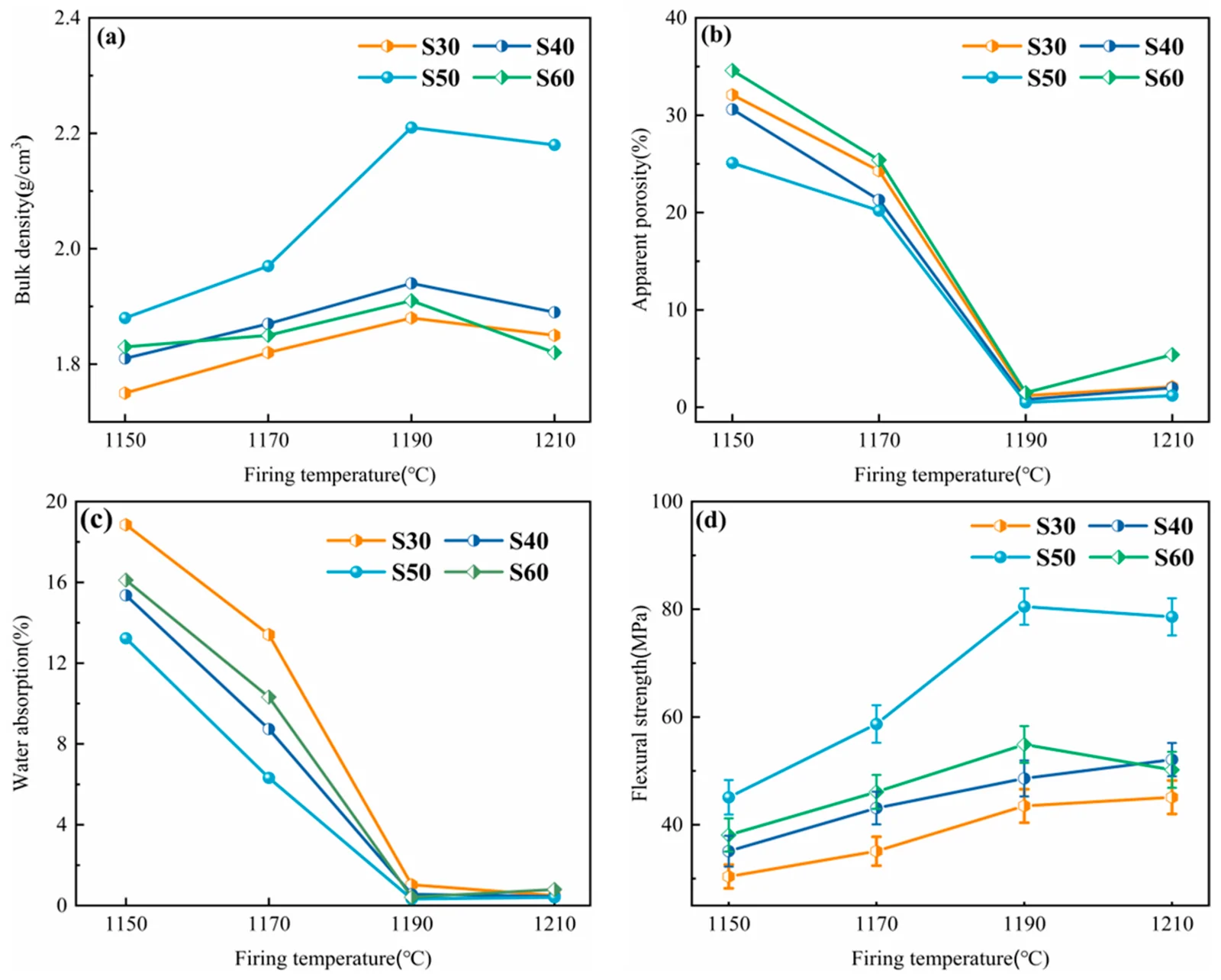
Variations in the physical and mechanical properties of the ceramics with tailings contents at different temperatures: (**a**) bulk density, (**b**) apparent porosity, (**c**) water absorption, (**d**) flexural strength.

The physical and mechanical properties (i.e., bulk density, apparent porosity, water absorption, and flexural strength) of the sintered tailings pyroxene-based ceramics as a function of sintering temperature and tailings content are illustrated in Figure 9. As shown in Figure 9a–c, specimens sintered below 1170 °C present a low bulk density and an apparent porosity higher than 20%, indicative of inadequate densification of the ceramic matrix. With the increase in sintering temperature, the bulk density of the ceramic samples increases markedly, reaching a maximum of 2.21 g/cm^3^ for sample S50 at 1190 °C. Meanwhile, both apparent porosity and water absorption drop to nearly zero, verifying that sintering temperature dominates the densification behavior of the as-prepared ceramics.

Combined with the microstructural evolution of pores in terms of quantity and morphology, it is demonstrated that the ceramics achieve optimal comprehensive physical and mechanical properties at this sintering temperature. A further temperature increases to 1210 °C leads to a slight reduction in bulk density, which is attributed to the excessive generation of liquid phase and the melting-induced deformation of pyroxene crystalline phases. Additionally, the temperature-dependent variation in flexural strength follows a trend consistent with that of the aforementioned physical properties. The flexural strength of each sample was obtained from five parallel specimens. Data points in Figure 9d represent the average values, and error bars denote the standard deviation, reflecting the experimental variability of the measurements. A relatively small standard deviation is observed for most samples, indicating good repeatability of the bending test results. Elevated temperatures facilitate liquid phase formation and ion diffusion, enabling more metal ions to dissolve into periclase and subsequently convert into augite with enhanced mechanical robustness. When the tailings dosage reaches 50 wt%, the flexural strength of the ceramic reaches 80.5 MPa, as illustrated in Figure 9d. Notably, its overall physical and mechanical properties comply with the performance specifications for porcelain tiles specified in the Chinese National Standard GB/T 4100-2015 [34] (flexural strength ≥ 35 MPa, water absorption ≤ 0.5%), demonstrating the great potential of tailings for high-value utilization in the ceramic industry.

**Figure 10:**
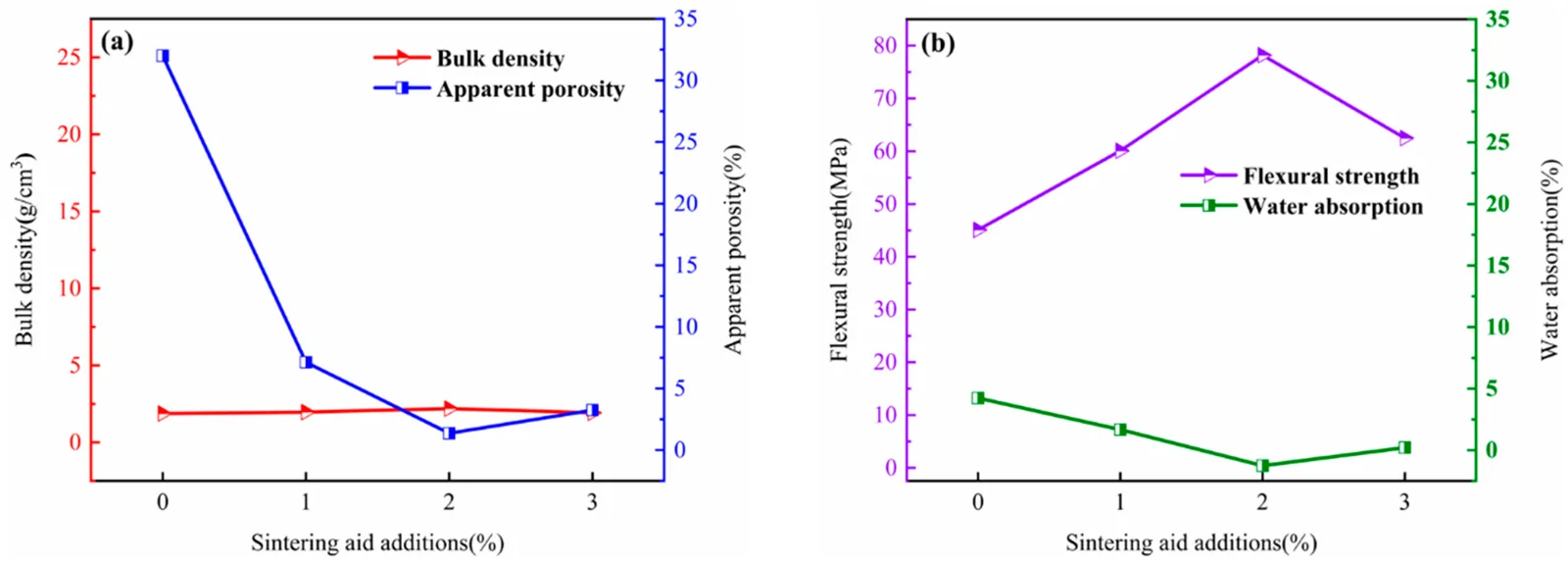
Variations in the physical and mechanical properties of the ceramics as a function of sintering aid addition: (**a**) bulk density and apparent porosity; (**b**) water absorption and flexural strengths.

The effect of sintering aid content on the comprehensive properties of ceramics sintered at 1150 °C is presented in Figure 10. At a low sintering aid dosage of 1 wt%, the ceramic specimen retains a relatively high apparent porosity. This phenomenon demonstrates that the limited amount of liquid phase generated within the ceramic matrix fails to promote sufficient mass transport, thereby resulting in a low densification degree. When the sintering aid dosage is increased to 2 wt%, both the apparent porosity and water absorption of the specimen decrease to nearly zero, accompanied by a flexural strength of 78.2 MPa. This indicates that the introduced sintering aid effectively promotes liquid-phase formation in the matrix, which significantly facilitates the densification process. Such a superior densification state is further verified by the microstructural characteristics in Figure 8: the specimen with 2 wt% sintering aid exhibits isolated circular closed pores and the highest bulk density among all groups. Nevertheless, a further increase in the sintering aid dosage to 3 wt% induces degradation in the mechanical and physical properties of the ceramics. This performance deterioration can be attributed to excessive liquid-phase generation. The superfluous liquid phase prematurely seals the open pores in the early sintering stage, trapping residual gas inside the matrix and hindering the elimination of internal pores, which ultimately suppresses ceramic densification. In summary, the optimal dosage of sintering aid can effectively expand the feasible firing temperature window and accelerate the densification kinetics of ceramic materials.

### 3.4. Leaching Behavior of Tailings Pyroxene-Based Ceramics

As discussed above, the tailings pyroxene-based ceramics exhibit excellent physical and mechanical properties. However, the magnetic separation tailings typically contain a certain amount of hazardous elements, such as heavy metals (Zn, Cu, Pb, and As), and thus, the environmental risk assessment of the tailings ceramic materials is crucial. A leaching toxicity test was performed to compare the environmental safety of the original magnetic separation tailings and the sintered ceramics, with results presented in Table 5. Compared with the tailings, the leached concentrations of Cu, Zn, Pb and As in the sintered ceramic products are further reduced far below the limit thresholds specified in Chinese National Standard GB/T 5085.3-2007 [35], highlighting the superior environmental safety performance of tailings-based ceramic materials. The research results by Pi et al. [36] and Guo et al. [37] indicate that extensive cation substitution occurs at the M1 and M2 lattice sites within the pyroxene phase (general formula: M_2_M_1_T_2_O_6_), which can effectively immobilize heavy metals ions into the crystal lattice and reduce the risk of environmental leaching. Combined with the leaching results of the pyroxene-based ceramics prepared in this study, it can be inferred that this phenomenon may also benefit from the isomorphous substitution characteristic of pyroxene group minerals. This demonstrates that pyroxene-based slag-derived ceramics possess great potential and feasibility for fabricating environmentally friendly ceramic materials.

From the above results and discussions, it can be seen that magnetic separation tailings can promote the sintering process of ceramics, and their optimal performance parameters and leaching toxicity fully meet the requirements of Chinese porcelain tiles standards and environmental risks. Table 6 shows the differences in mechanical properties and environmental safety between traditional ceramics and other slag-derived ceramics, as well as the magnetic tailings ceramics used in this study. Compared to traditional ceramics, the solid waste-based ceramics have good performance and environmental safety, demonstrating the great potential for the application of metallurgical solid waste in the ceramic industry.

## 4. Conclusions

In this study, a novel pyroxene-based ceramic was prepared using magnetic separation tailings derived from synergistic treatment of converter slag and copper slag as the main raw materials. The effect of tailings content, sintering temperature and sintering aid additions on the phase composition, microstructure evolution, physical and mechanical properties, and environmental safety of the prepared ceramics were systematically investigated. The main conclusions are summarized as follows:

(1) With increasing tailings content, the crystalline phase of the ceramic gradually evolves from a mixed quartz–pyroxene phase to a pyroxene phase. At a tailings content of 50 wt%, the pyroxene phase exhibits the strongest and sharpest diffraction peaks, indicating that sufficient crystallization and grain growth of pyroxene were achieved during the sintering process.
(2) Elevated tailings content and firing temperature both promoted pyroxene grain growth and liquid-phase formation. The pore morphology transformed from narrow, interconnected pores to rounded, closed pores, significantly enhancing ceramic densification. At a tailings content of 50% and firing temperature of 1190 °C, the ceramic achieved a water absorption of 0.33% and flexural strength of 80.5 MPa, fully satisfying the performance requirements for porcelain tiles specified in relevant Chinese National Standards.
(3) The addition of sintering aid effectively lowers the sintering temperature, accelerates liquid phase formation and grain growth, promotes the early formation of uniform closed pores, thereby further improving ceramic densification.
(4) Compared with the raw tailings, the leaching toxicity of sintered pyroxene-based ceramics is further reduced and complies with the specified regulatory limits. This indicates that fabricating green, safe and high-performance slag-derived ceramic products from industrial solid waste possesses broad application prospects.

## Figures and Tables

**Table 1:** Chemical compositions of the raw materials (wt.%).

	CaO	SiO_2_	MgO	Al_2_O_3_	Fe_2_O_3_	Na_2_O	LOI
Tailings	61.68	24.48	6.30	6.16	1.39	/	/
Kaolin	0.07	46.57	0.14	38.25	0.73	0.61	13.63
Quartz	/	≥99.50	/	/	/	/	/
Talc	0.26	59.99	31.56	1.53	1.11	0.02	5.52
Albite	0.37	67.93	0.18	19.86	0.07	11.24	0.35

**Table 2:** Experimental design for the effect of tailings content on ceramic properties (wt.%).

Batches	Tailings	Kaolin	Quartz	Talc
S30	30	28	17	25
S40	40	26	14	20
S50	50	24	11	15
S60	60	22	8	10

**Table 3:** Experimental design for the effect of sintering aid addition on ceramic properties (wt.%).

Batches	Tailings	Kaolin	Quartz	Talc	Albite
NS1	49.50	23.76	10.89	14.85	1
NS2	49.01	23.52	10.77	14.70	2
NS3	48.52	23.28	10.66	14.54	3

**Table 4:** Chemical compositions of the ceramics in designed formulation (wt.%).

	CaO	SiO_2_	MgO	Al_2_O_3_	Fe_2_O_3_	Na_2_O
S30	19.28	55.67	10.41	13.69	0.95	/
S40	25.60	50.65	9.34	13.40	1.01	/
S50	31.87	45.66	8.29	13.10	1.08	/
S60	38.11	40.71	7.23	12.81	1.14	/
NS1	31.54	45.88	8.23	13.17	1.07	0.12
NS2	31.22	46.11	8.15	13.24	1.06	0.23
NS3	30.90	46.33	8.07	13.31	1.05	0.35

**Table 5:** Leaching test results of the tailings and representative ceramic sample S50 (mg/L).

	Cu	Zn	Pb	As
Tailings	80.56	8.31	1.93	0.95
S50	0.320	0.112	0.047	0.013
Toxicity thresholds	100	100	5	5

**Table 6:** Comparison of the basic properties of the prepared ceramic in this work with other waste-derived ceramics.

Sources	Materials	Substitution Ratio/wt%	Sintering Temperature/°C	Major Phases	Flexural Strength (MPa)	Leaching Test Results
Ref. [38]	Clay Feldspar Quartz	/	1250	Mullite Quartz	<70	/
Ref. [24]	Steel slag	40	1210	Pyroxene	143	/
Ref. [30]	Reduced copper slag + 4%TiO_2_	40	1175	Anorthite	81.7	Cu 0.116 (mg/L) Zn 0.067 (mg/L) As 0.009 (mg/L) Pb 0.006 (mg/L)
Ref. [31]	Reduced copper slag	45	1175	Anorthite Diopside	78.3	Cu 0.084 (mg/L) Zn 0.055 (mg/L) As 0.008 (mg/L) Pb 0.006 (mg/L)
Ref. [39]	Ferrochrome slag Tundish slag	20 ferrochrome; 85 tundish slag	1240; 1100–1120	Pyroxene	114.52; 124.61	Cr 0.15%; Mn 0.98%
Ref. [40]	Red mud	50	1130	Pyroxene Anorthite	115.88	Na^+^ 0.33%; K^+^ 0.10%
Our work	Magnetic separation tailings	50	1190	Pyroxene	80.5	Cu 0.320 (mg/L) Zn 0.112 (mg/L) As 0.013 (mg/L) Pb 0.047 (mg/L)

## Data Availability

The original contributions presented in this study are included in the article. Further inquiries can be directed to the corresponding authors.
